# Early life malaria exposure and academic performance

**DOI:** 10.1371/journal.pone.0199542

**Published:** 2018-06-22

**Authors:** Ninja Ritter Klejnstrup, Julie Buhl-Wiggers, Sam Jones, John Rand

**Affiliations:** 1 Department of Food and Resource Economics, University of Copenhagen, Copenhagen, Denmark; 2 Copenhagen Business School, Copenhagen, Denmark; 3 Department of Economics, University of Copenhagen, Copenhagen, Denmark; Universidade Nova de Lisboa Instituto de Higiene e Medicina Tropical, PORTUGAL

## Abstract

Malaria is a major cause of morbidity and mortality in sub-Saharan Africa. It is also a dynamic contributor to poverty through its effects on children’s cognitive development. This paper examines the degree to which malaria in early childhood impacts on educational achievement in later childhood. The substantial decline in malaria in the region over recent years allows an assessment of its impact to be made. Focusing on Tanzania, we combine data from the Malaria Atlas Project and the 2010–2014 Uwezo household surveys (N = 246,325). We relate the district-level risk of malaria in a child’s year of birth to his/her performance in tests of acquired cognitive skills (literacy and numeracy). For causal identification, we rely on differences across districts in the pace of decline in malaria prevalence occurring over the last 15 years. We control for time-invariant district level, age, birth cohort and survey year effects, as well as district-level trends and individual and household-specific factors. In addition, we use sibling variation in birth-year exposure to malaria to strengthen our identification. A ten percentage-point decrease in malaria prevalence in birth year is associated with a 0.06 standard deviation (p = 0.000) increase in English literacy achievement. This estimate is comparable in magnitude to education intervention programs with very large effects. Our results are robust to a large number of sensitivity analyses. We find no statistically significant effects of birth-year malaria exposure on attainments in numeracy and Kiswahili, and we argue that this is probably attributable to strong ceiling effects in these test scores. We conclude that in Tanzania malaria is an important factor in geographical variation in English literacy. This indicates that malaria is a significant public health challenge to educational achievement in this country, and probably in other regions with malaria.

## Introduction

Malaria infection in childhood has been associated with persistent cognitive impairment [1–3]. The association is particularly strong with severe forms of malaria resulting from infection with the *Plasmodium falciparum* parasite, including cerebral malaria, malaria with seizures, and severe malarial anemia [4–6]. However, uncomplicated [7–10] and asymptomatic malaria [11] have also been negatively associated with performance in cognitive tests and academic achievement in prospective case-control [8], cohort [7,10] or cross-sectional comparison studies [9,11]. Further, randomized controlled trials (RCTs) in which prophylaxis or treatment were given to school-aged children have found that preventive measures can lead to improvements in cognitive or academic performance [12,13].

These findings suggest that post-malarial cognitive impairment represents a significant public health challenge to educational achievement [5,14] and may also, as a consequence, be an obstacle to the alleviation of poverty in regions where malaria is present. However, previous studies suffer from a number of methodological weaknesses that limit our ability to draw firm conclusions regarding causality and the magnitude of the problem. Most are based on small samples that are not representative on a national scale. Moreover, despite controlling for an observed list of potential confounders, case-control, cohort and cross-sectional comparison studies potentially involve omitted variables bias.

A separate recent body of literature has explored longer-term effects of childhood malaria using either temporal variation in climatic variables [15,16] or implementation of malaria eradication programs [17–23] as exogenous sources of variation in exposure. Most of these studies have looked at outcomes in adulthood, and most find that exposure to malaria in childhood depresses income in adulthood. Some have also looked at adult literacy and educational attainment, with more mixed results, especially in the latter case. For example, in Mexico, Hoyt Bleakley found evidence that exposure to malaria in childhood led to lower income and a lower probability of being literate in adulthood but had no effect on years of schooling [17]. This is consistent with long-term income consequences of malaria being driven primarily by effects on cognitive abilities (as formed in childhood) rather than the mere quantity of schooling. Meanwhile, to our knowledge only one of the studies from this body of literature (focusing on Zambia) looks directly at skill formation during childhood [23]. This study concludes that children exposed to low but resurgent malaria prevalence in infancy perform worse in cognitive tests at age six than children who are exposed to higher malaria prevalence from birth. Ruling out other explanations, the authors argue that this is probably due to the first group’s failure to form partial immunity in infancy–an immunity which would protect them in the context of rising prevalence.

In this study, we explore how exposure to malaria in early childhood affects performance in literacy and numeracy tests in later childhood in a context where prevalence is not on the rise. We employ an indirect approach relating the risk of contracting malaria in early childhood, defined as the district-level prevalence of malaria in the child’s year of birth, to their performance on benchmarked literacy and numeracy tests when they are of school age. In view of the phased and cumulative nature of malaria interventions in Tanzania, we do not depend on a unique exogenous policy intervention to obtain causal identification. Rather, we rely on the observed temporal and spatial variation in malaria risk associated with the rapid decline in prevalence over the last 15 years. We use repeated cross-sectional data on test scores, collected at the household level, which are representative at both national and district levels. This enables us to control for time-invariant district level, age, birth cohort and survey year effects, as well as individual and household-specific factors. To our knowledge this is the first study to use nationally representative longitudinal data to estimate the impact of early life malaria exposure on academic performance in school-age children in Africa. The richness of our data allows us to address many of the potential weaknesses of previous studies, and as a consequence we are able not only to estimate the effect of early life malaria exposure on academic performance in mid to late childhood, but also to shed light on the importance of malaria exposure in explaining geographical inequalities in academic performance in the context of Tanzania.

## Methods

### Study population and data sources

The data used for this study refer to children resident in mainland Tanzania aged 7–14 during the period 2010–2014. We combine national- and district-level representative data on academic performance among children with district-level estimates of *Plasmodium falciparum* parasite rates (PfPR) for each year from 2000 to 2015. The latter represent direct metrics of malaria risk, which we calculate based on pixel level estimates from the Malaria Atlas Project (MAP) [24] and pixel level population estimates from the WorldPop Project [25,26]. The MAP data consist of continuous predictions of *Plasmodium falciparum* parasite rates among 2 to 10 year-olds (hereafter simply referred to as PfPR) at 5 × 5 km spatial resolution. These are generated using a spatiotemporal Bayesian model combining (i) PfPR estimates of 27,573 spatially and temporally unique population clusters obtained from hundreds of community-based or national cross-sectional surveys from across the African continent, (ii) an optimized set of environmental covariates, and (iii) modeled coverage of Insecticide-Treated bed Nets (ITN), Artemisinin-based Combination Therapies (ACT) and Indoor Residual Spraying (IRS). In the modeling process, survey-based estimates of PfPR are adjusted by age, season and type of diagnostic (rapid diagnostic testing/microscopy), and the modelling of ITN coverage includes socio-demographic covariates [24]. Of the 27,573 population clusters 2,678 are from Tanzania (see S4 Fig for a map of clusters).

**Fig 1:**
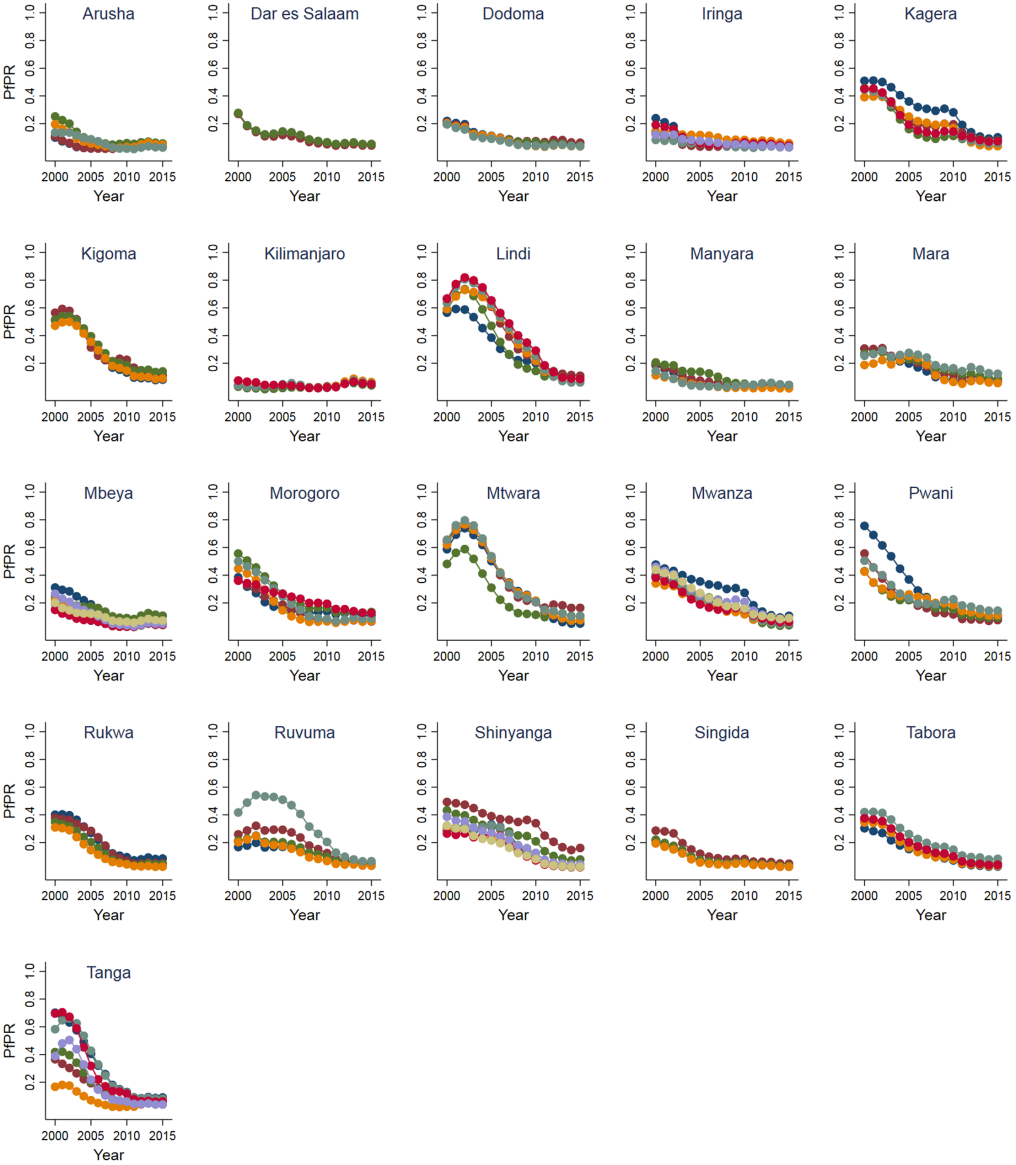
Developments in Plasmodium falciparum parasite rates for districts within regions of Tanzania, 2000–2015. Each graph represents a region, and each line a district within a region, with 2002 boundaries obtained from the Global Administrative Unit Layers initiative.

The WorldPop data consist of predictions of population density at 1x1 km spatial resolution. These predictions are generated using a non-parametric model in which census data are combined with land cover information, climatic data, elevation data, observed lights at night, information on networks of roads and waterways, large water bodies, and protected area delineations [25]. In the case of Tanzania, population grids are available for 2000, 2005, 2010 and 2015. We estimate intermediate years by taking the natural logarithm of the WorldPop estimates, linearly interpolating the missing years, and finally taking the exponential for the individual years. To arrive at annual district-level estimates of PfPR, we aggregate population data to pixels that correspond to the resolution of the MAP data. For each pixel we then multiply PfPR and the population count and overlay the resulting raster with 2002 district boundaries obtained from the Global Administrative Unit Layers initiative [27]. District-level averages are then calculated by summing over all pixels that fall within a district and dividing by the district population size. Fig 1 illustrates the spatial and temporal variation in PfPR for districts within regions of Tanzania during the period 2000–2015. A recent study concluded that the large drop in prevalence between 2000 and 2015 was due primarily to unprecedented levels of intervention coverage during this period [24].

Data on children’s educational performance come from the 2010–2014 Uwezo cross-sectional surveys for Tanzania [28]. The data are representative at the district level, and cover children aged 7–16, both in and out of school. In the African context, this level of within-country coverage is unparalleled, and it provides a valuable opportunity to analyze developments over time and across space. The tests are designed to measure basic skills in English and Kiswahili literacy as well as numeracy. The numeracy test has six incremental levels: Number recognition, counting, magnitude comparison, addition, subtraction and multiplication. The literacy tests have five incremental levels: Letter recognition, reading words, reading paragraphs, reading a story and comprehension of the story read. For each test, children who fail to pass any of the levels are awarded a score of one. The other children are awarded a score equal to the highest level passed plus one. The difficulty of the tests is fixed and corresponds to the level expected after two years of completed schooling. The tests were developed by a panel of experts from the Tanzanian Institute of Education, the University of Dar Es Salaam, practicing teachers and experts in the subject area. They were refined in consultation with the Ministry of Education and Vocational Training to ensure that they reflect the national second grade curriculum.

The pooled test score dataset, which covers the surveys undertaken annually from 2010 to 2014, includes a total of 199,767 households and reflects a coverage rate of 79 percent relative to the survey design. From the pooled dataset we exclude children born before 2000, as this is the first year for which we have malaria data. Further, as we wish to control for birth-year fixed effects in our analysis (see below), we exclude children born in 2007, as this birth year is only found in the 2014 survey. This gives us a final pooled sample size of 246,325 children. Table 1 presents summary statistics for the main variables included in this study, split by low/high malaria prevalence districts.

**Table 1:** Summary of district by birth cohort means, by PfPR category.

	Low prevalence PfPR districts		High prevalence PfPR districts		Difference in means	
	Mean	Standard deviation	Mean	Standard deviation	Mean	Standard deviation
*Individual*:						
Age	9.634	1.876	9.630	1.880	0.004	0.008
Female (share)	0.506	0.500	0.505	0.500	0.001	0.002
Birth order	2.516	1.543	2.643	1.643	-0.127***	0.006
Currently enrolled (share)	0.895	0.306	0.879	0.327	0.016***	0.001
Never enrolled (share)	0.071	0.256	0.087	0.281	-0.016***	0.001
Highest completed grade	2.974	1.877	2.715	1.810	0.259***	0.008
English score	0.615	1.669	0.187	1.554	0.428***	0.007
Numeracy score	-0.274	2.106	-0.621	2.122	0.347***	0.009
Kiswahili score	0.129	1.859	-0.178	1.848	0.307***	0.007
*Household*:						
Poor (share)	0.430	0.495	0.460	0.498	-0.030***	0.002
Ultra-poor (share)	0.128	0.334	0.117	0.322	0.011***	0.001
Maternal education—none (share)	0.183	0.387	0.228	0.420	-0.045***	0.002
Maternal education—primary (share)	0.728	0.445	0.698	0.459	0.030***	0.002
Maternal education—secondary (share)	0.034	0.180	0.026	0.160	0.008***	0.001
Maternal education—post secondary (share)	0.015	0.122	0.010	0.099	0.005***	0.000
Household size	6.779	3.211	7.043	3.383	-0.264***	0.013
*District*:						
Night lights per capita	0.002	0.003	0.002	0.002	0.000***	0.000
Birth-year PfPR	0.152	0.085	0.404	0.144	-0.252***	0.000
Survey-year PfPR	0.056	0.027	0.098	0.041	-0.042***	0.000

Notes: Summary statistics pertain to the sample of birth cohorts born between 2000 and 2006, both years included. Low prevalence districts are districts with PfPR below median (0.322) in 2000. Low prevalence districts are districts with PfPR below median (0.322) in 2000. The standard deviation of the pooled (both low and high PfPR) distributions is 1.627 (English), 1.860 (Kiswahili) and 2.121 (numeracy).

*** denotes significance at the 1% level. The average district size is 8923 Km2 (minimum 80 km2 and maximum 55.816 km2)

The last data source is the National Centers for Environmental Information (NOAA) [29], which provides data on night-time light density from 1992 to 2013 at pixel level. Since data on economic activity over time and at the sub-country level is very limited or non-existent in low income contexts, night-time light density has been widely used in recent years to investigate variation in development outcomes [30].

Since the study is based entirely on existing, publicly available, anonymized datasets, no ethical approval has been sought.

### Procedures and statistical analysis

We investigate the causal link between infection by malaria in early childhood and academic performance in later childhood (7–14 years of age). Thus, the outcomes of interest are performance in numeracy and English and Kiswahili literacy tests.

As a result of the nature of the Uwezo tests, raw test scores are not comparable across ages and survey years. Specifically, since the tests are not age-dependent, the median test scores increase with age (see S1–S3 Figs). By implication, the same raw score may be indicative of median performance among 7 year-olds but be well below median performance for 14 year-olds. Similarly, as there is some variation in the difficulty of tests across the survey years, the same raw test score may be indicative of a different standard of performance for individuals of the same age in different years. To increase the comparability of test score interpretations across individuals in the pooled sample, we therefore center raw test scores by age and year by subtracting the median score of all children of the same age tested in the same year from individual test scores. The centered test scores (hereafter referred to simply as the test scores) then indicate deviations from the median performance of children of the same age in the same year. We chose this transformation rather than the common approach of standardizing test scores by mean and standard deviations, because the test score distributions are highly skewed, and the median is a better measure of central tendency than the mean in skewed distributions.

Our main predictor variable is PfPR at the district level in a child’s birth year. We choose birth-year prevalence as a measure of the risk of contracting malaria in early childhood rather than an average prevalence over a given age range in order to avoid arbitrarily assigning specific weights to risks experienced at distinct ages in early childhood. It should be noted, however, that birth-year prevalence is in fact strongly correlated with the arithmetic mean of the risk experienced at each age prior to school entry (correlation coefficient 0.810). This essentially means that we would not expect to see different results if, instead, we were to use an average prevalence over a given age range (results using average prevalence for the three-year period covering the year before, of and after birth are available in the S1 Table). The decision to use birth year rather than any other year in early childhood was motivated by the facts (i) that for the majority of children, this is when the district-level risk is highest, and (ii) that pre-natal period and infancy are fundamental periods of brain development and hence most likely to be associated with the highest risk of cognitive impairment [31]. Importantly, however, given the strong correlation of district-level PfPR between years, we are not claiming to identify the specific effect of birth-year exposure but rather use birth-year PfPR as a proxy for early life exposure.

The analysis proceeds as follows. First, we present the crude association between birth-year PfPR and test scores graphically, plotting average test scores for groups defined by 0.1 intervals of birth-year PfPR. Second, we assess the relationship between birth-year PfPR and test scores in a multivariate regression framework. In addition to malaria exposure, we expect individual test scores to be determined by individual and household-specific factors, contextual factors in early childhood, and contextual factors in the survey year. To account for these multiple sources of variation, our baseline specification is as follows:

$$y_{ihjkt}=\;\alpha_{0}+\beta {\text{PfPR}}_{kj}+X_{it}^{'}\gamma +X_{ht}^{'}\delta +\theta x_{jk}+\lambda_{jt}+\lambda_{k}+\varepsilon_{it}$$

where *y* is the centered test score of interest, *i* indexes individuals, *h* indexes the household to which the child belongs, *k* indexes the child’s birth cohort (i.e. year or birth), *j* indexes the district of residence, and *t* indicates the year of the survey. On the right-side of the model, in addition to the main explanatory variable of interest (PfPR) and residual error (*ε*), we include five main groups of controls. These are: (1) individual factors (denoted *X*_*i*_, which are her gender, birth order and a set of dummy variables for child age); (2) household factors (denoted *X*_*h*_, which are the size of the household, three dummy variables capturing the highest level of education of the mother’s education, and two dummy variables for socio-economic status, based on ownership of assets); (3) district-level nightlights per capita in the child’s year of birth (denoted *x*_*jk*_), which represents a time-varying measure of economic development at the district level; (4) joint survey year and district fixed effects (denoted *λ*_*jt*_); (5) birth year given by the penultimate term. Note that the observed covariates were chosen based on the full set of information available in the datasets across all survey rounds (i.e. no controls were initially included but subsequently dropped). Also, fixed effects for each survey year are implicitly included since they are a linear combination of birth year and age.

To estimate the above specification, we rely on ordinary least squares (OLS) methods, treating the various sets of unobserved group-level effects (i.e. birth cohort, district by survey year etc.) as fixed effects. This is implemented in Stata v15.1 using the user-written command *reghdfe* [32]; a full set of replication files is also available in S1 File. We prefer OLS as its properties are well established and it constitutes a highly robust estimator for conditional means, regardless of the underlying distribution of the data [33]. OLS estimators naturally extend to incorporate multiple fixed effects, and we adopt this treatment of the unobserved terms since random or mixed effects approaches typically require some assumptions to be made about the structure of correlation between the random effects and other covariates. Fixed effects estimators, by contrast, impose no such assumptions and are thus expected to be consistent over a broader range of conditions. Furthermore, we found that the large size of the dataset and the multiple unobserved terms included in our model meant that alternative mixed effects (maximum likelihood) estimators frequently failed to converge.

In the calculation of standard errors for our estimates, we adopt a two-way clustering procedure. Here we follow Abadie and Wooldridge [34] who identify two primary and separate reasons for using clustered standard errors. The first is a sample design rationale, which captures the notion that if there are clusters in the population that are not represented in the sample, one should cluster errors at the level of such ‘missing’ clusters. In our case, these are the PSUs (villages) rather than administrative districts, since all districts are covered by the surveys. The second rationale for clustering arises when some aggregate of units, rather than individuals (or the units of observation), is assigned collectively to a treatment. In our case, following our definition of PfPR, this is the district by cohort birth year. We consider that both these sources of clustering are potentially relevant and, thus, we apply two-way clustering by the PSU and the district by cohort birth year. Note that this delivers more conservative results than clustering by either factor separately.

We recognize that our baseline specification may not capture the full range of factors that potentially confound the relationship between malaria and test scores. For example, high PfPR districts may have been systematically favored in the allocation of health interventions, and if these interventions had independent effects on children’s cognitive development this may have biased our results. To examine whether this source of bias is material, we estimate the relationship between PfPR and test scores using a household fixed effects estimator. This amounts to replacing the set of observed household covariates (*X*_*h*_) with a general household-specific term, which is also treated as a fixed effect in the estimation. Because children born in different years living in the same household are likely to be less differentially affected by changes that occur between their birth years than children born in different birth years in different households, this should reduce (but may not entirely eliminate) such potential bias. For instance, a mother who receives health care information for her second child may use that information to improve care for her first child. Another mother who has a child in the same year as the first mother’s first child, but does not have a subsequent child, on the other hand, may not receive the improved information at all, and hence may not improve her child’s care. Therefore, substantial variations in estimates with and without these household fixed effects may indicate that some part of the partial correlation between malaria reduction and test scores is driven by other district-specific time-varying factors (such as childhood development interventions). On the other hand, the discovery of similar estimates would strengthen confidence in the assumption that districts have not experienced such differential paths.

In addition to the baseline and household fixed effects specifications, we undertake a series of robustness checks. First, we construct a pseudo-panel at the level of birth cohorts within districts [17,35]. The average outcomes in this panel will be a result of district-birth-year average individual and contextual factors, most of which are absorbed by district and birth-year fixed effects, which we include in a multivariate linear regression. This approach will deliver consistent estimates of the relationship between malaria and test scores provided that we assume that district-specific shocks or developments in the birth-year period covered by our analysis do not affect children born in different years differentially on average. Note that, under the assumption that seasonality in births is constant across the birth years considered, this approach also alleviates concerns that results are confounded by the facts that in the main analysis, (1) individuals born days apart, but in different years, are assigned different risks of malaria; and (2) individuals born within the same year but in seasons with different levels of malaria prevalence are assigned the same risk of malaria.

Next, we address the fact that we have chosen a transformation of data which is not standard in educational research. This may lead to a concern that our results are sensitive to the particular choice of transformation. To address this, we revert to the raw test scores and create a set of binary (dummy) variables, taking a value of 1 if the child scores above each specific increment. So, a child with the lowest test score (none correct) receives a zero for each of these dummy variables; and a child with the highest test score (all correct) receives a one for each. For each dummy variable we re-run our household fixed effects model, which amounts to a linear probability model in each case. Note that this method is similar to the first stage of the approach, set out in [36], used to consistently estimate parameters of an ordered logit model in the presence of fixed effects. The point is that by looking at the various incremental scores separately we take into account the ordinal nature of the raw test scores and allow for unequal differences in ability at each increment (i.e. the difference in ability between children receiving 1 and 2 may be larger or smaller than the difference between children receiving 3 and 4).

The next two robustness checks are a response to the fact that from the modelled PfPR data (Fig 1) it appears that in some districts PfPR increased between 2000 and 2002. While this increase may be an empirical reality, we cannot rule out the possibility that it is due to the specifics of the modelling behind the estimates. To deal with this concern, we test the sensitivity of our results to (i) dropping individuals from districts with an initial rise in PfPR, and (ii) dropping individuals born before 2002. The final robustness check is needed because a reduction in malaria which affects a given cohort in its birth year could also affect adjacent cohorts and thereby introduce imprecision in the measure of birth-year malaria exposure. To address this concern, we restrict the sample to individuals born several years apart who are therefore likely to have experienced different disease environments. In particular, we drop individuals born between 2002 and 2004.

## Results

**Fig 2:**
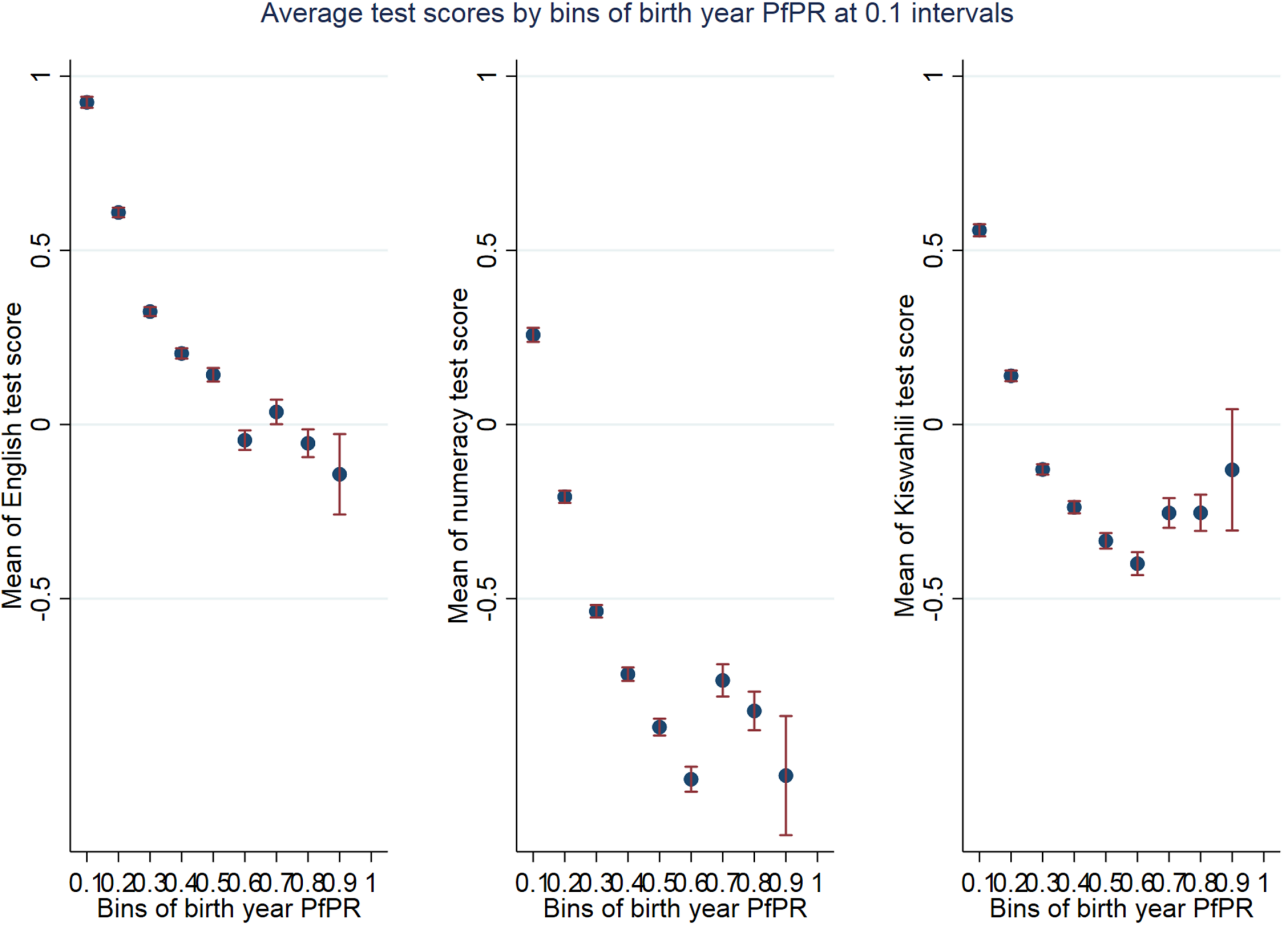
The relation between average academic performance and birth-year PfPR. Note that test scores are averaged for individuals with birth-year PfPR falling within the same 0.1 interval.

Fig 2 shows that in all three tests the average performance of Tanzanian children decreases as birth-year PfPR increases (for a visualization of the spread of test scores by 0.1 intervals of birth-year PfPR, see violin plots in S5–S7 Figs).

Columns (1), (4) and (7) in Table 2 report the estimated crude bivariate association between birth-year PfPR and test scores for English, numeracy and Kiswahili, respectively. In keeping with the graphical evidence, a significant negative association can be seen between children’s birth-year exposure to malaria and their performance in all tests. Columns (2), (5) and (8) present estimates from the multivariate model described above, which includes all controls. For English literacy, the relationship between birth-year malaria prevalence and test scores is reduced in magnitude but remains statistically significant. For numeracy and Kiswahili the coefficient magnitude is reduced even further, becoming insignificant. These results are robust to ignoring sampling weights (results available upon request). Columns (3), (6) and (9) show the results from estimations using household fixed effects. Note that the number of observations for these regressions is smaller than it is for the other regressions, as only households with at least two children aged 7–14 are included. These results confirm those obtained in the previous multivariate models. This suggests that potential bias from omitted birth year by district-level factors is at most negligible.

**Table 2:** Estimates of the relation between test scores and birth-year PfPR.

	(1)	(2)	(3)	(4)	(5)	(6)	(7)	(8)	(9)
	English	English	English	Numeracy	Numeracy	Numeracy	Kiswahili	Kiswahili	Kiswahili
Birth-year PfPR	-2.003***	-0.903***	-0.758***	-2.286***	0.083	0.051	-2.064***	-0.036	0.057
	(0.122)	(0.161)	(0.206)	(0.170)	(0.239)	(0.254)	(0.143)	(0.203)	(0.230)
Controls	No	Yes	Yes	No	Yes	Yes	No	Yes	Yes
HH fixed effects	No	No	Yes	No	No	Yes	No	No	Yes
Observations	246,325	246,325	149,262	246,325	246,325	149,262	246,325	246,325	149,262
R-squared	0.042	0.182	0.722	0.034	0.194	0.712	0.034	0.239	0.715

Notes: All regressions are estimated using OLS. Dependent variable: Individual test score centered with the survey year and age-specific median. Standard errors appear in parenthesis and are clustered by village and district-by-cohort. In columns 2, 3, 5, 6, 8 and 9 the estimates are corrected for: individual and household characteristics (age, gender, birth order, household size, maternal education and wealth), birth year, year, district and district-by-year fixed effects as well as birth-year district-level economic development (measured as night-time lights). Population weights applied.

***, ** and * denote significance at the 1, 5 and 10% levels, respectively.

For English, the estimated coefficient on birth-year malaria prevalence under the preferred model (Table 2, column 2) is -0.903 (SE 0.161; p = 0.000). The magnitude of the implied relationship between birth-year malaria and English literacy can be grasped by considering the implied effect of going from a birth-year prevalence rate equal to the average of “high prevalence” districts (0.404; see Table 1) to a rate equal to the average of the “low prevalence” districts (0.153; see Table 1). This is equivalent to reducing birth-year PfPR by 0.251, which translates to a 0.227 point expected increase in the test score. Equivalently, this represents an increase of 0.140 standard deviations of the pooled test score distribution (pooled SD = 1.627, see Table 1).

In Table 3 we present the results of a series of robustness checks for the effect on English (equivalent results for the effects on numeracy and Kiswahili can be seen in S3–S8 Tables). Row 1 shows the results from estimating the alternative specification using a pseudo-panel of cohort level averages. Rows 2, 3 and 4 address the validity of the malaria data. Finally, rows 5 to 9 show the results obtained by estimating our specification using different definitions of the outcome variable. None of these raises concerns that the negative association between birth-year PfPR and performance on the English achievement test is fragile.

**Table 3:** Robustness of English achievement.

			Full sample		Household FE	
Model	Description	Outcome	N	Estimate	N	Estimate
(1)	Cohort-level results	Cohort average test score	2,542	-1.002***		-
				(0.167)		-
(2)	Reduced cohorts	Individual centered test score	160,167	-1.193***	68,329	-1.047***
				(0.196)		(0.277)
(3)	Reduced districts	Individual centered test score	186,141	-1.235***	113,299	-1.580***
				(0.245)		(0.288)
(4)	Only youngest and oldest cohorts	Individual centered test score	129,056	-0.929***	42,821	-0.973***
				(0.180)		(0.308)
	*Competency cutoff*					
(5)	Cutoff 1	Dummy variable (letters or higher = 1)	246,325	0.057*	149,262	0.070
				(0.032)		(0.049)
(6)	Cutoff 2	Dummy variable (words or higher = 1)	246,325	-0.132***	149,262	-0.123**
				(0.033)		(0.050)
(7)	Cutoff 3	Dummy variable (paragraph or higher = 1)	246,325	-0.245***	149,262	-0.243***
				(0.043)		(0.059)
(8)	Cutoff 4	Dummy variable (story or higher = 1)	246,325	-0.227***	149,262	-0.202***
				(0.043)		(0.064)
(9)	Cutoff 5	Dummy variable (full competencies = 1)	246,325	-0.206***	149,262	-0.168***
				(0.041)		(0.059)

Notes: All regressions are estimated using OLS. Dependent variable: Individual test score centered with the survey year and age-specific median. Standard errors appear in parenthesis and are clustered by village and district-by-cohort. In rows 2–9 estimates are adjusted for: individual and household characteristics (age, gender, birth order, household size, mother's educational level and wealth), birth year, year, district and district-year fixed effects as well as birth year district-level economic development (measured as night-time lights). Population weights applied.

***, ** and * denote significance at the 1, 5 and 10% levels, respectively.

Row 1 shows the results obtained by estimating a pseudo-panel of cohort level averages. Row 2 shows the results obtained by dropping individuals born before 2002. Row 3 shows the results obtained by dropping individuals from districts with an initial rise in PfPR. Row 4 shows the results obtained by dropping individuals born between 2002 to 2004. Rows 5 to 9 show the results of estimating the effect of birth-year PfPR at each specific increment of the test scores.

## Discussion

We have shown that birth-year district-level malaria prevalence negatively predicts performance in English literacy tests among children of primary school age. Our estimates suggest that a reduction of average birth-year prevalence in districts with above-median prevalence to that in districts with below median prevalence would increase average English test scores by 0.144 standard deviations (0.121, when household fixed effects are factored in). At first glance, this may seem to be a small effect, but it needs to be seen in the context of the generally low sensitivity of test scores to interventions, even at the school level [37,38]. A recent meta-analysis based on 77 randomized experiments, with a total of 111 treatment arms, has compared the effects of different categories of school-based intervention on learning in developing country primary schools across different learning outcomes [38]. Our estimates of the effect of malaria on English test scores are comparable to interventions with the largest mean effect sizes. This includes, for example, interventions based on student/teacher performance incentives (mean effect size 0.089 standard deviation), contract or volunteer teachers (mean effect 0.101 SD), reduction in class size, ability grouping or smaller learning groups within classes (mean effect 0.117 standard deviation), teacher training classes (mean effect 0.123 SD), and treatments with computers or instructional technology (mean effect 0.150 SD).

The same meta-analysis found that school-based health and nutrition interventions generally had limited or statistically insignificant mean effects. In view of this, it seems reasonable to conclude that the estimated effect of malaria on performance in English tests is material. The same conclusion can be drawn when we consider the educational achievement gap between areas of high and low malaria prevalence. For instance, the difference in average test scores between districts at or above the 95th percentile of the PfPR distribution in 2000 and districts at or below the 5th percentile of the PfPR distribution was 1.156 points. The difference in average birth-year PfPR in those districts is 60.4 percentage points. Thus, our effect estimate of -0.783 to -0.934 corresponds to around 40 to 50 percent of the test score gap between these districts. While this seems to be a very large effect magnitude, we need to bear in mind that a 107 to 128 percent decrease in malaria risk would be required for the test score of a child to increase by just one point relative to the median child in his/her age group. The largest decrease in district-level PfPR during the last 15 years was 93 percent. Hence, while birth-year PfPR represents an important factor in explaining why children in high malaria prevalence areas perform worse in English literacy tests than children in low malaria prevalence areas, it is only a part of the overall explanation for differences in academic achievement between children. Given the relatively large effect on English literacy, it is perhaps surprising that we did not find an overall effect on numeracy or Kiswahili. However, in this context it should be noted that in a recent validation of the Uwezo tests undertaken in Kenya it was found that the tests in numeracy and Kiswahili discriminate only to a limited extent between children’s skill levels [39]. The reason is that these tests do not have items of difficulty that fully span the different ability levels in early grades. They only span difficulties at the lower end of the ability distribution. In effect, the insignificant effect on numeracy and Kiswahili literacy scores does not necessarily mean that early life malaria exposure has no effect, but rather reflects strong ceiling effects in the numeracy and Kiswahili test scores (see S8 Fig). One way to assess this possibility is to estimate our specification using only children for whom ceiling effects are small. We therefore re-estimated our baseline and household fixed effect models using only children who were 7–9 years old (See S1–S3 Figs for the age-specific test score distributions).

Table 4 shows that when we confine our attention to children aged 7–9 years old, we find that birth-year PfPR has strong negative effects on academic achievement across all three test scores. This supports the argument that strong ceiling effects in the numeracy and Kiswahili test scores are likely to explain the insignificant results. This in turn suggests that the effect lies at the upper end of the ability distribution, as ceiling effects would not influence the results if the effects were in the lower end of the ability distribution.

**Table 4:** Main results using only children aged 7 to 9 years.

	(1)	(2)	(3)	(4)	(5)	(6)
	English	English	Numeracy	Numeracy	Kiswahili	Kiswahili
						
Birth-year PfPR	-1.709***	-1.747***	-0.739**	-1.449**	-0.682***	-0.752
	(0.262)	(0.433)	(0.286)	(0.591)	(0.221)	(0.566)
Observations	119,611	31,658	119,611	31,658	119,611	31,658
R-squared	0.187	0.745	0.222	0.771	0.210	0.742
Household FE	No	Yes	No	Yes	No	Yes

Notes: All regressions are estimated using OLS. Dependent variable: Individual test score centered with the survey year × age-specific median. Standard errors appear in parenthesis and are clustered by village and district-by-cohort. All estimates are adjusted for: individual and household characteristics (age, gender, birth-order, household size, mother's educational level and wealth), birth year, year, district and district-by-year fixed effects as well as birth-year district-level economic development (measured as night-time lights). The sample is restricted to children between 7–9 years old. Population weights applied.

*** and ** denote significance at the 1 and 5% levels, respectively.

We found that our results are insensitive to a large number of robustness analyses. Nonetheless, four additional issues should be mentioned. The first relates to the malaria prevalence data, which do not allow us to ascertain whether any given child actually has malaria. Here we rely on an “intention-to-treat” approach, assuming that children in higher prevalence areas are more likely to contract malaria. However, if there is systematic difference in the relative probability of malaria infection between high and low achieving children across high and low PfPR areas, this could create a bias leading either to over- or underestimation of the effect of malaria on academic performance. The averaging process in the dynamic pseudo-panel approach should, however, eliminate the individual-level measurement error giving rise to this type of bias. Hence the fact that our results are robust to this approach suggests that we should not be concerned that individual-level measurement error is leading us to under- or overestimate results.

The second issue relates to the potential of “selective mortality” to confound results. That is, assuming that death from malaria is more likely among children who are disadvantaged in terms of the determinants of learning (e.g. cognitive abilities or parental income levels), it is possible that the reduction in malaria prevalence led to a change in birth cohort composition producing a larger share of children with less in the way of prerequisites of learning. This would bias our results towards zero, as older cohorts, born in periods of higher malaria prevalence, would on average be doing better than they would have if more disadvantaged children had survived. Thus, if selective mortality occurred, our findings may have underestimated the true effect of malaria exposure on academic performance.

Third, all of our analyses assume that a child’s district of current residence is also the district of his/her early childhood residence. This introduces the possibility that “selective migration” biased our results. For example, if it is more common for the more able children to move from areas of high prevalence to areas of low prevalence during childhood, we may be underestimating their earliest childhood malaria exposure. However, excluding regions with above median in-migration from our analysis does not change our results (results available on request).

The fourth issue is that we cannot adjust for district-specific cohort effects, for example, due to selective allocation of health interventions. Addressing this by comparing results with and without household fixed effects, we found strong correspondence. As children within the same household are likely to be less differentially affected by changes that occur between their birth years than children from different households, we take the strong correspondence to indicate that results are not likely to be confounded by district-specific cohort effects. This reasoning fails, however, if older siblings are not affected by changes that occur after their year of birth in ways that influence their potential for learning in later life–for example, if the only elements of postnatal care interventions that affect learning are infant-specific elements such as promotion of exclusive breastfeeding, or if mothers do not in fact implement recommended practices such as immunization to older children. In response to this concern, we would point out that the two largest national child health programs, both of which expanded during our study period, were the Expanded Program on Immunization (EPI) and the Integrated Management of Childhood Illness (IMCI). EPI already had very high national coverage in 1999 (over 80 percent) [40], and therefore it is less likely that there would be systematic differences in immunization between children born in different years after 2000 within the same household. EPI was expanded in 2002 to include a DPT-HepB vaccine, and this could, potentially, have resulted in a difference between cohorts born before and after this year. However, our results are robust to excluding these cohorts (see Table 3). It is true that the IMCI program, covering only 50 percent of districts in 2004 [41], is more likely to be a source of confounding. However, our main results are robust to the inclusion only of districts that had implemented IMCI prior to 2000 (results available on request).

In short, although we cannot rule out the possibility that our results are subject to bias, there is no evidence that the bias is, or is likely to be, severe. Consequently, our findings corroborate the existing evidence that exposure to malaria in early life materially impairs academic performance. We conclude that in the Tanzanian context, at least, early childhood malaria exposure represents a significant public health challenge with real implications for academic performance.

## Supporting information

S1 Fig Distributions of raw English test scores by age. (TIF)

S2 Fig Distributions of raw numeracy test scores by age. (TIF)

S3 Fig Distributions of raw Kiswahili test scores by age. (TIF)

S4 Fig Map of malaria data clusters. (PNG)

S5 Fig Violin plots of centered English test scores by bins of birth-year PfPR. (TIF)

S6 Fig Violin plots of centered numeracy test scores by bins of birth-year PfPR. (TIF)

S7 Fig Violin plots of centered Kiswahili test scores by bins of birth-year PfPR. (TIF)

S8 Fig Raw test score distributions. (PNG)

S1 Table Results using moving average birth-year PfPR. (PDF)

S2 Table Main results with covariates. (PDF)

S3 Table Robustness: Cohort level estimates. (PDF)

S4 Table Robustness: Reduced cohorts. (PDF)

S5 Table Robustness: Reduced districts. (PDF)

S6 Table Robustness: Only young and old cohorts. (PDF)

S7 Table Robustness: Numeracy competence cutoffs. (PDF)

S8 Table Robustness: Kiswahili competence cutoffs. (PDF)

S1 File Complete set of replication do-files. (ZIP)
