# Supplementary figures and images for: Early life malaria exposure and academic performance
Source: PLoS One. 2018 Jun 22;13(6):e0199542. doi: 10.1371/journal.pone.0199542 (PMC6014671; doi:10.1371/journal.pone.0199542)

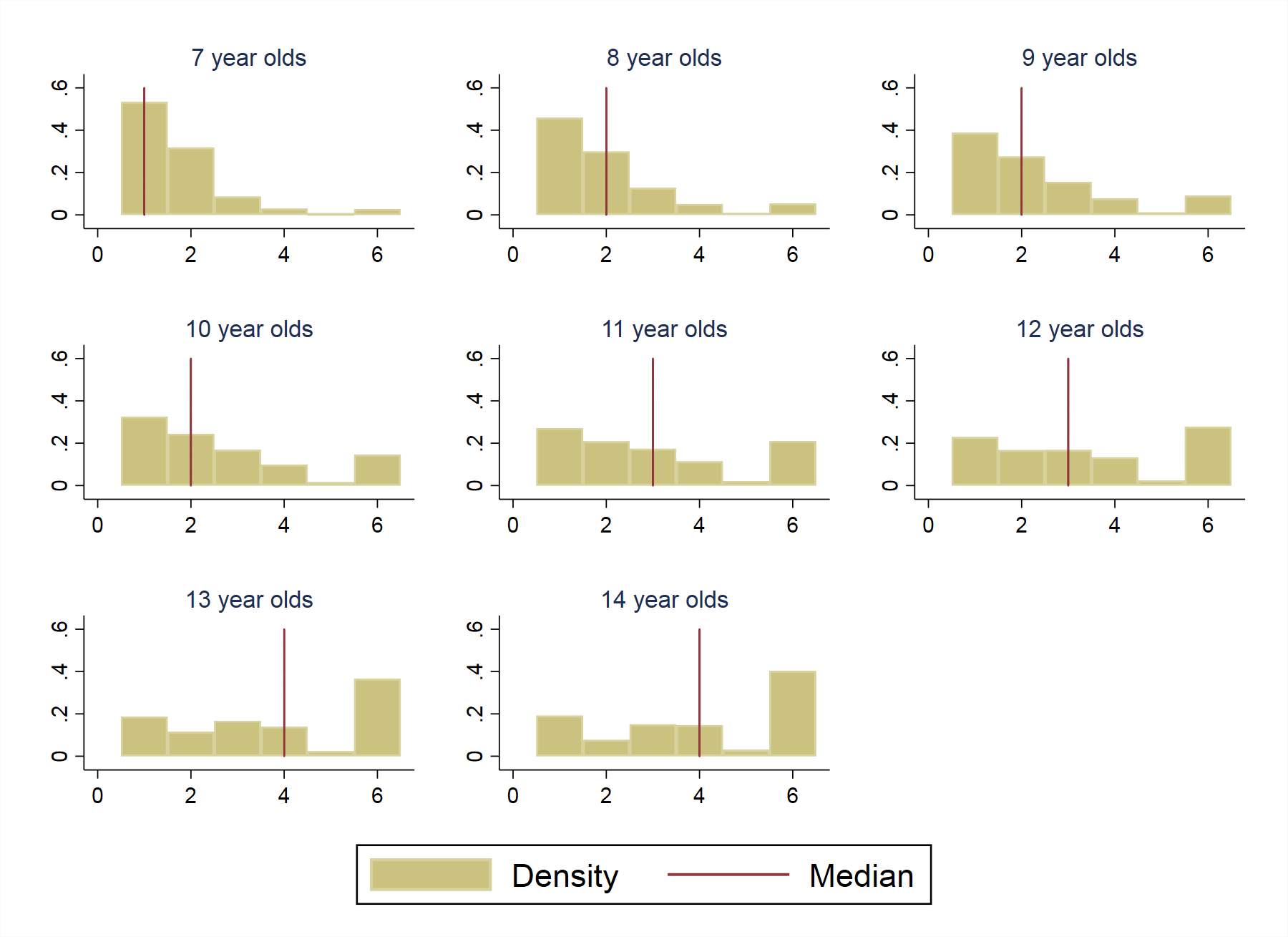

Supplement: S1 Fig — (TIF) [file pone.0199542.s001.tif]

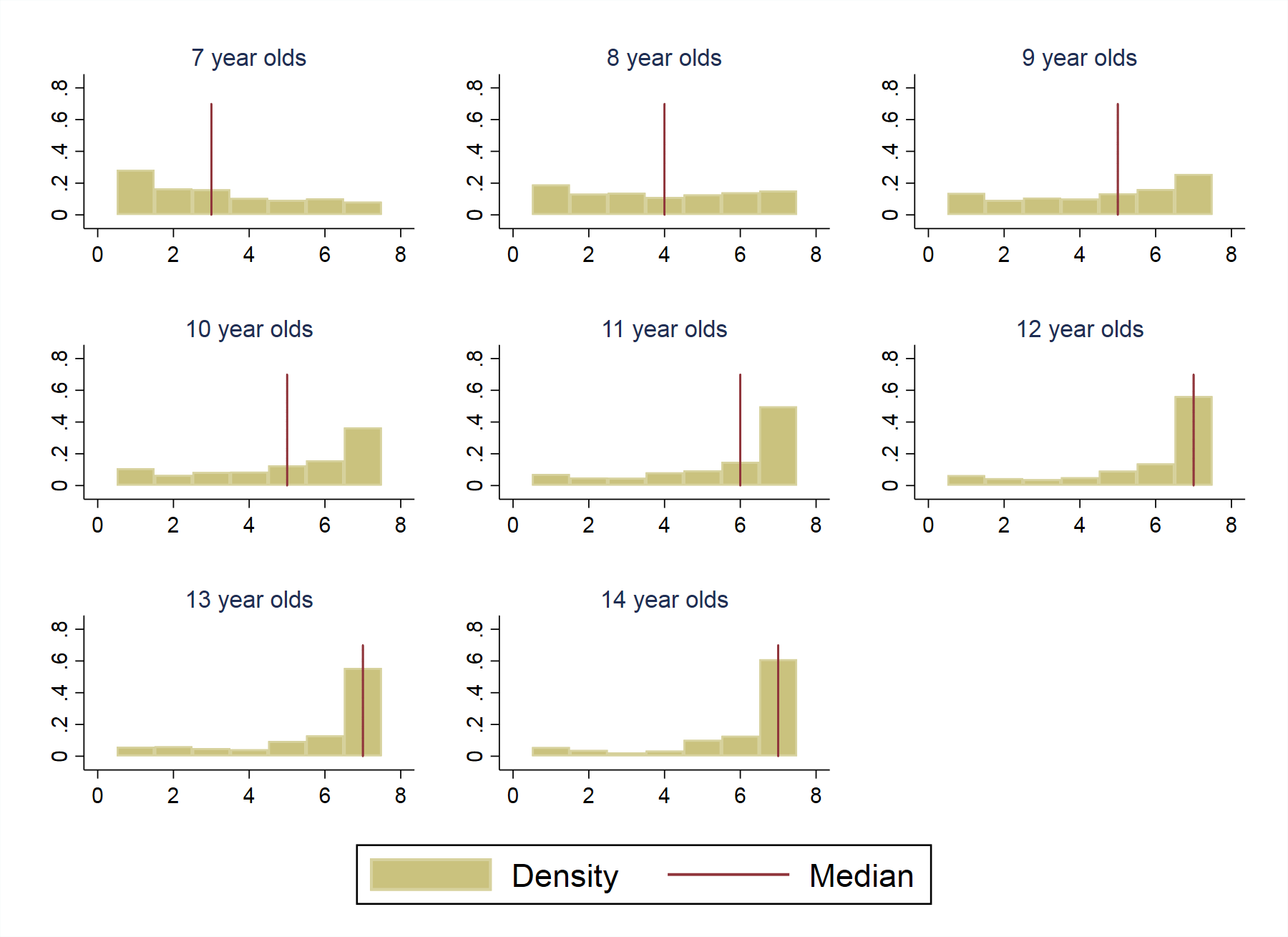

Supplement: S2 Fig — (TIF) [file pone.0199542.s002.tif]

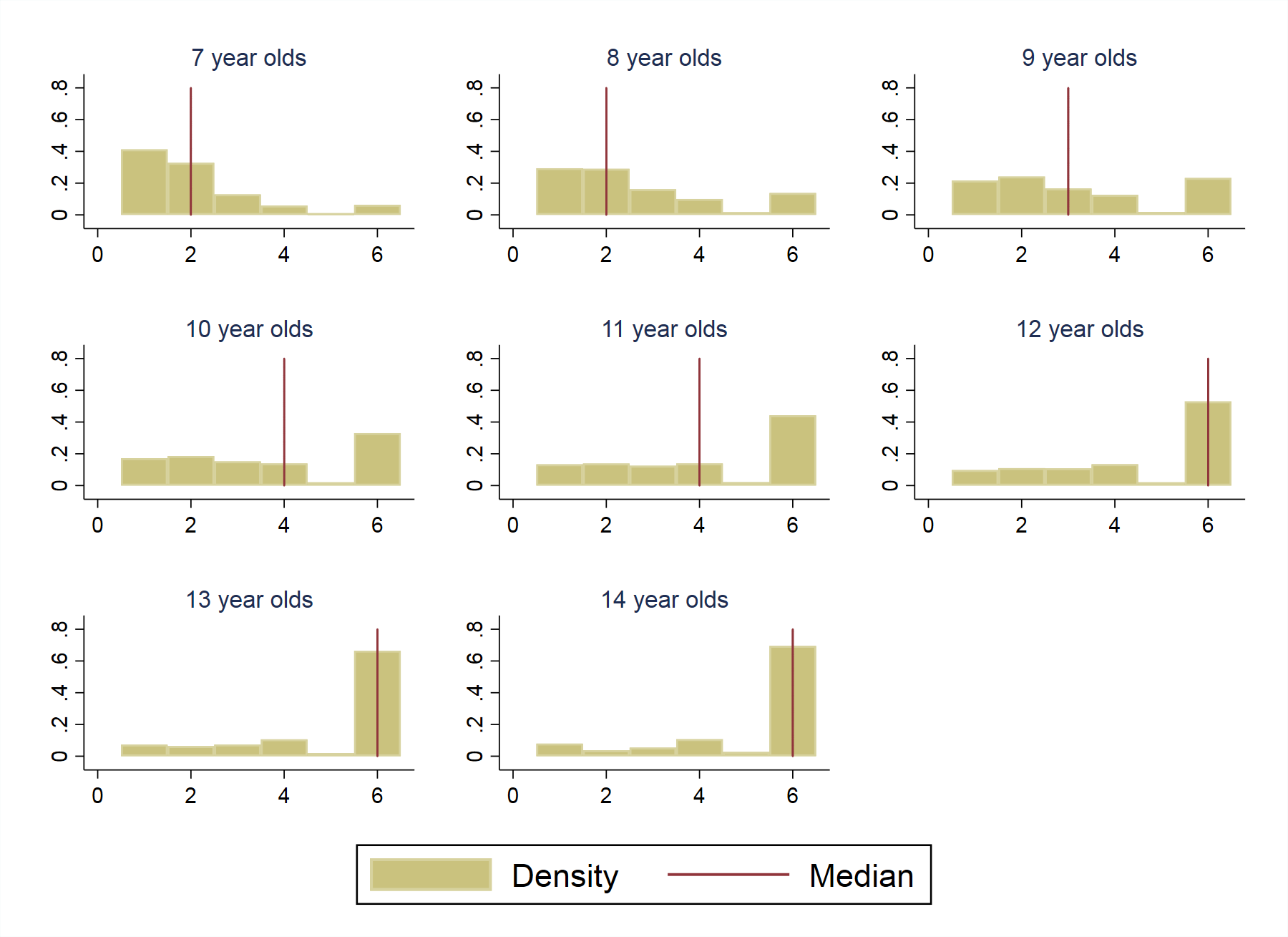

Supplement: S3 Fig — (TIF) [file pone.0199542.s003.tif]

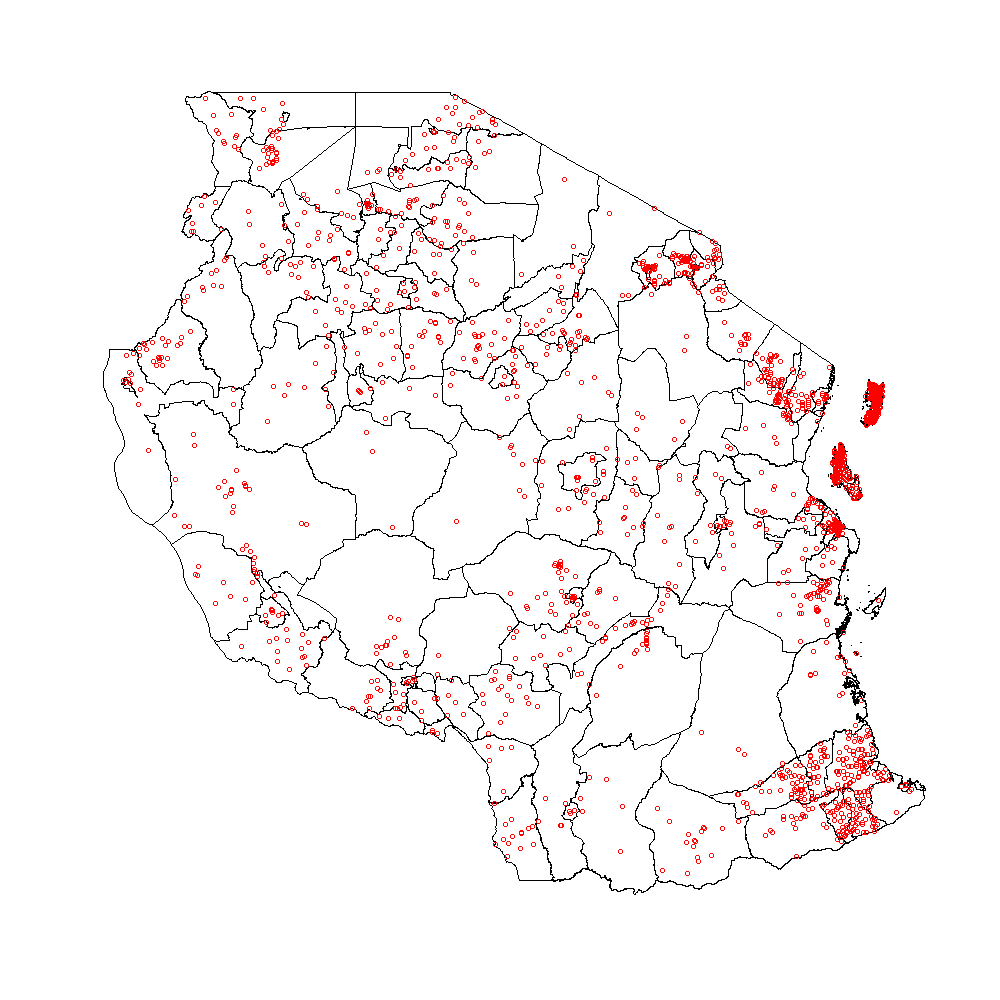

Supplement: S4 Fig — (PNG) [file pone.0199542.s004.png]

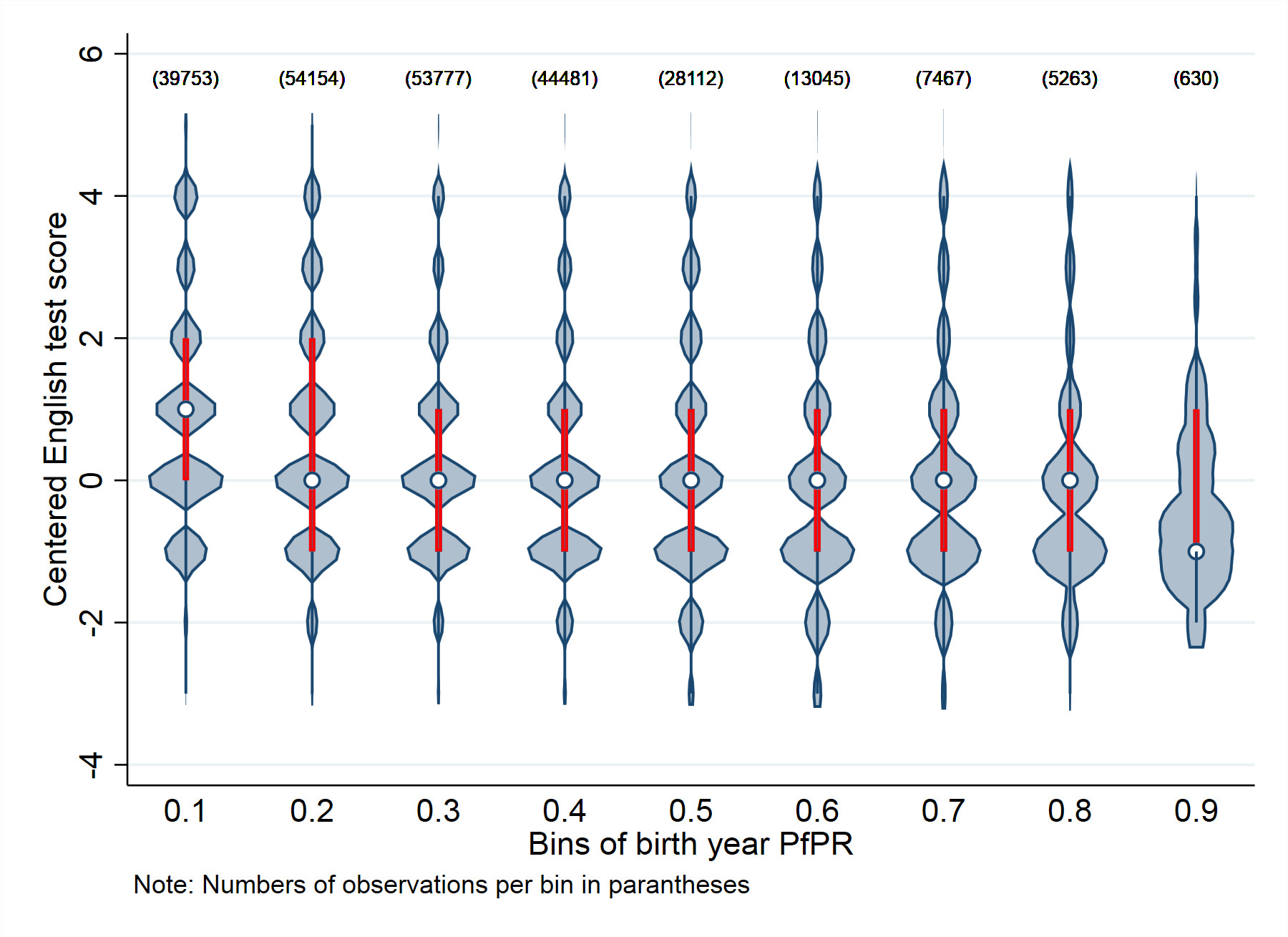

Supplement: S5 Fig — (TIF) [file pone.0199542.s005.tif]

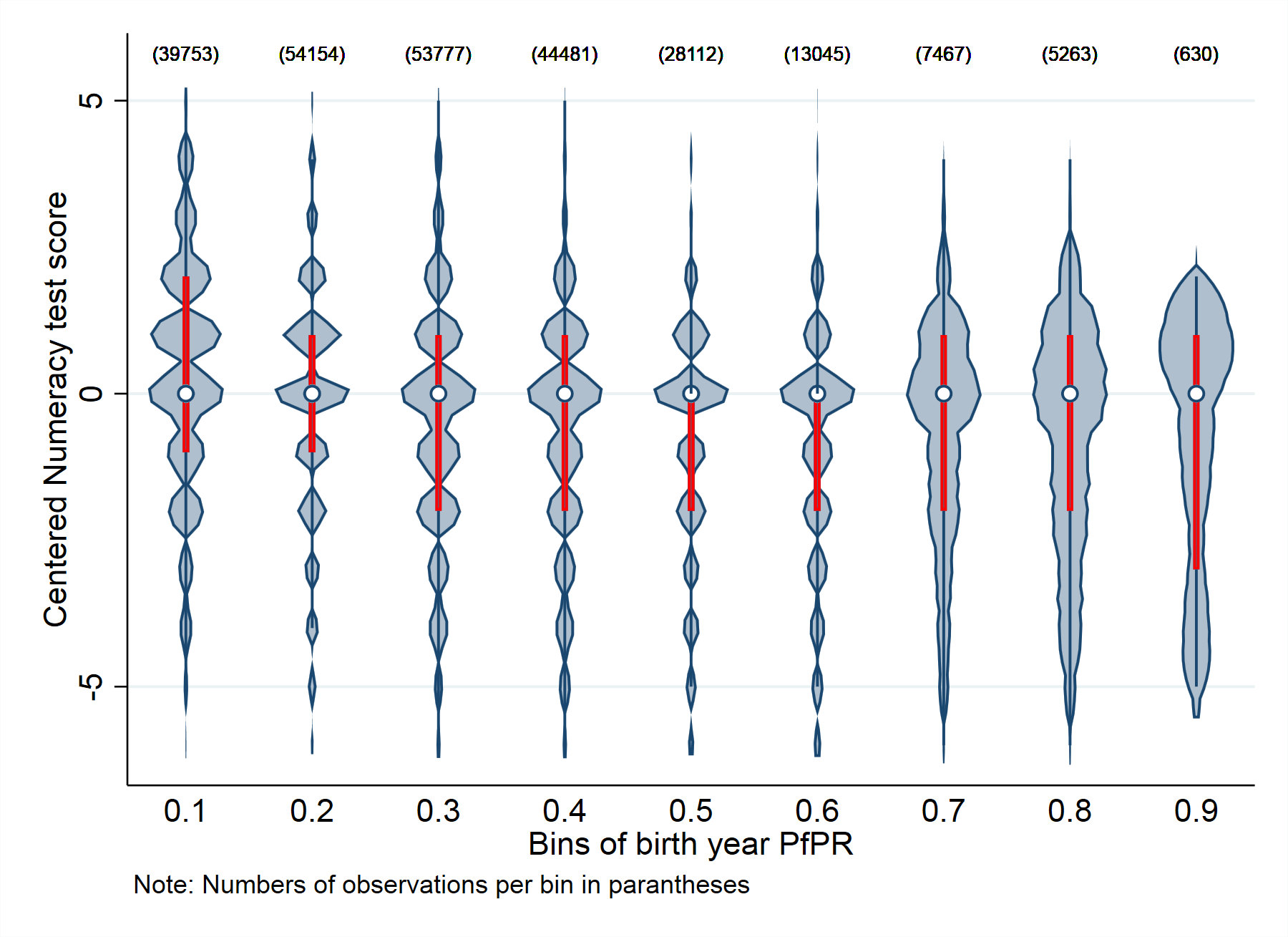

Supplement: S6 Fig — (TIF) [file pone.0199542.s006.tif]

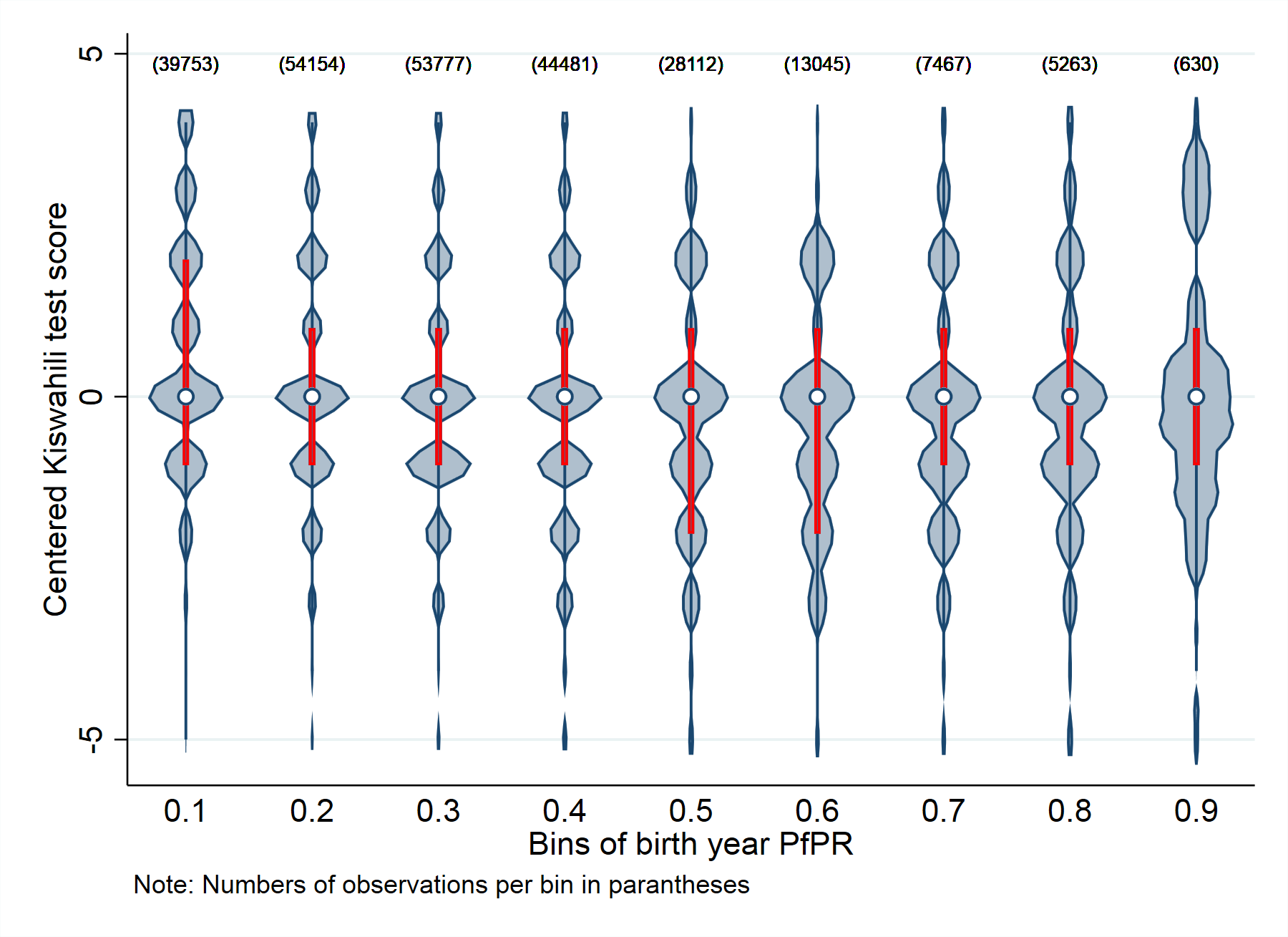

Supplement: S7 Fig — (TIF) [file pone.0199542.s007.tif]

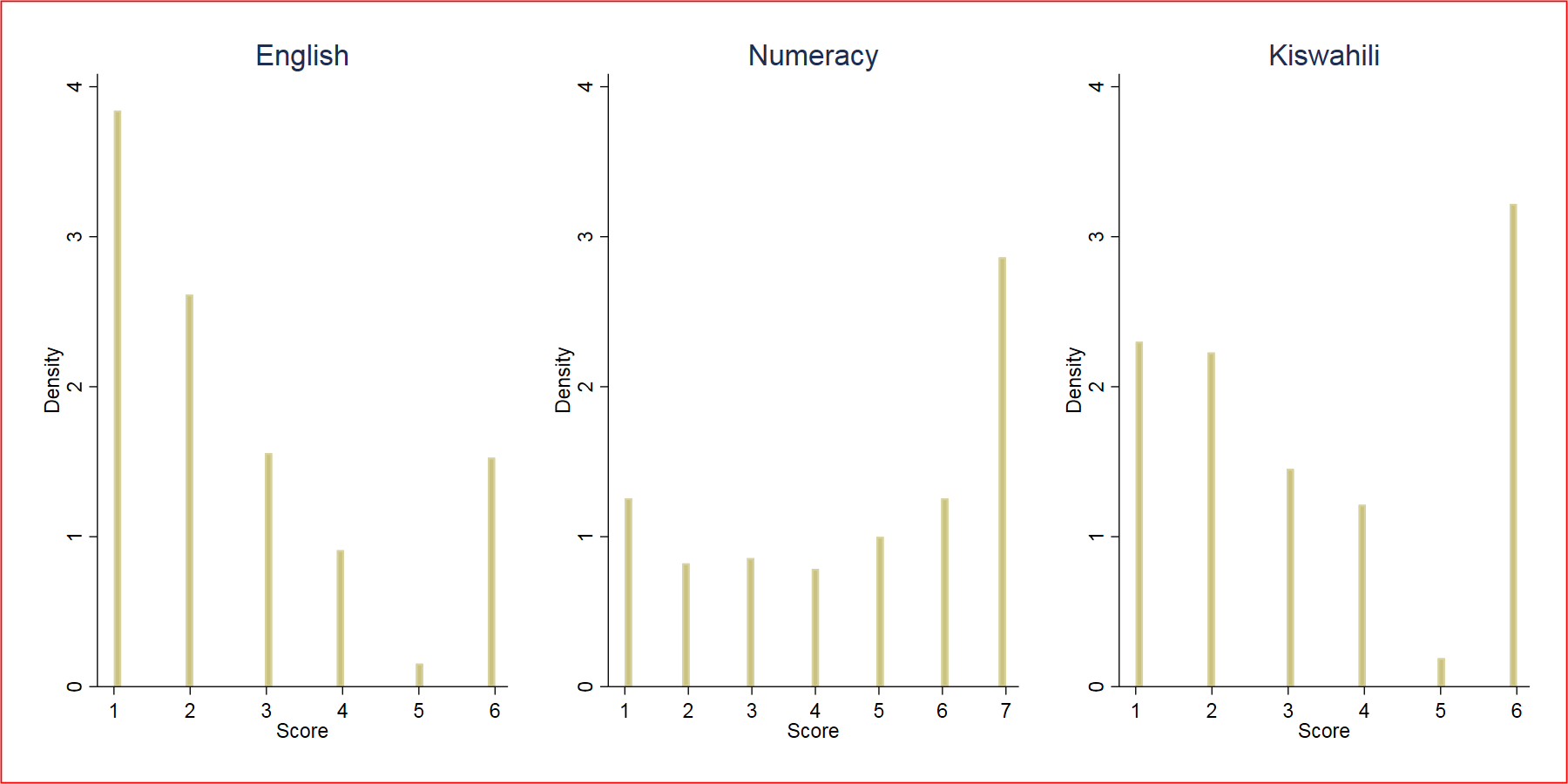

Supplement: S8 Fig — (PNG) [file pone.0199542.s008.png]
