# Supplementary material for: Early life malaria exposure and academic performance
Source: PLoS One. 2018 Jun 22;13(6):e0199542. doi: 10.1371/journal.pone.0199542 (PMC6014671; doi:10.1371/journal.pone.0199542)
Supplement: S1 Table — (PDF) [file pone.0199542.s009.pdf]

S1 Table: Results using moving average birthyear PfPR

	(1) English	(2) English	(3) Numeracy	(4) Numeracy	(5) Kiswahili	(6) Kiswahili
Moving average birth-year PfPR	-1.269***	-1.142***	0.305	0.412	0.170	0.284
	(0.199)	(0.262)	(0.340)	(0.362)	(0.227)	(0.314)
Observations	200,251	103,924	200,251	103,924	200,251	103,924
R-squared	0.190	0.741	0.190	0.726	0.260	0.738
Household FE	No	Yes	No	Yes	No	Yes

Notes: All regressions are estimated using OLS. Dependent variable: Individual test score centered with the survey year × age specic median. Standard errors appear in parathesis and are clustered by village and district-by-cohort. All estimates are adjusted for: individual and household characteristics (age, gender, birthorder, household size, mother's educational level and wealth), birth year, year, district and district-by-year fixed effects as well as birthyear district-level economic development (measured as nighttime lights). The variable of interest is a three year moving average of malaria PfPR around the birthyear. Population weights applied. *** and ** denotes significance at the 1 and 5 %-level, respectively.
