# Supplementary material for: Early life malaria exposure and academic performance
Source: PLoS One. 2018 Jun 22;13(6):e0199542. doi: 10.1371/journal.pone.0199542 (PMC6014671; doi:10.1371/journal.pone.0199542)
Supplement: S2 Table — (PDF) [file pone.0199542.s010.pdf]

S2 Table: Main results with covariates

	(1)	(2)	(3)
	English	Numeracy	Kiswahili
Birth-year PfPR	-0.903***	0.0827	-0.0363
	(0.161)	(0.239)	(0.203)
Female	0.105***	0.138***	0.183***
	(0.0101)	(0.0117)	(0.0111)
Birthorder	-0.00703	-0.00918	-0.0254***
	(0.00480)	(0.00631)	(0.00478)
Household size	-0.0144***	-0.0133***	-0.0102***
	(0.00227)	(0.00279)	(0.00236)
Maternal education (primary)	0.218***	0.444***	0.362***
	(0.0165)	(0.0211)	(0.0167)
Maternal education (secondary)	0.909***	1.090***	1.003***
	(0.0427)	(0.0440)	(0.0468)
maternal education (post secondary)	0.966***	0.803***	0.706***
	(0.0681)	(0.0693)	(0.0587)
Household wealth (Poor)	-0.292***	-0.366***	-0.330***
	(0.0149)	(0.0167)	(0.0146)
Household wealth (Ultra poor)	-0.421***	-0.610***	-0.517***
	(0.0204)	(0.0264)	(0.0214)
Night lights	86.21***	-83.65***	-70.77***
	(12.70)	(19.47)	(13.81)
Observations	246,325	246,325	246,325
R-squared	0.182	0.194	0.239

Notes: All regressions are estimated using OLS. Dependent variable: Individual test score centered with the survey year and age specific median. Standard errors appear in parathesis and are clustered by village and district-by-cohort. All estimates are corrected for: individual and household characteristics (age, gender, birthorder, household size, maternal education and wealth), birthyear, year, district and district-by-year fixed effects as well as birthyear district-level economic development (measured as nigthtime lights). Population weights applied. ***, ** and * denotes significance at the 1, 5 and 10 %-level, respectively.
