# Supplementary material for: Early life malaria exposure and academic performance
Source: PLoS One. 2018 Jun 22;13(6):e0199542. doi: 10.1371/journal.pone.0199542 (PMC6014671; doi:10.1371/journal.pone.0199542)
Supplement: S3 Table — (PDF) [file pone.0199542.s011.pdf]

S3 Table Robustness: Cohort Level Estimates

	(1)	(2)	(3)
	English	Numeracy	Kiswahili
Birth-year PfPR	-1.002***	-2.235***	-0.659**
	(0.167)	(0.267)	(0.271)
Observations	2,542	2,542	2,542
R-squared	0.664	0.691	0.641

Notes: All regressions are estimated using OLS. Dependent variable: Birthyear-by-district average test score. All regressions include cohort, age, year, district and district-by-year fixed effects as well as birth year district-level economic development (measured as nighttime lights). Standard errors clustered at the survey year-by-district-level appear in parenthesis. Averages are weighted by the square root of the number of observations used to compute them. *** and ** denotes significance at the 1 and 5 %-level, respectively.
