# Supplementary material for: Early life malaria exposure and academic performance
Source: PLoS One. 2018 Jun 22;13(6):e0199542. doi: 10.1371/journal.pone.0199542 (PMC6014671; doi:10.1371/journal.pone.0199542)
Supplement: S4 Table — (PDF) [file pone.0199542.s012.pdf]

S4 Table Robustness: Reduced cohorts

	(1)	(2)	(3)	(4)	(5)	(6)
	English	English	Numeracy	Numeracy	Kiswahili	Kiswahili
Birth-year PfPR	-1.193***	-1.047***	0.0720	0.447	0.108	0.243
	(0.196)	(0.277)	(0.342)	(0.393)	(0.237)	(0.333)
Observations	160,167	68,329	160,167	68,329	160,167	68,329
R-squared	0.190	0.754	0.191	0.746	0.269	0.754
Household FE	No	Yes	No	Yes	No	Yes

Notes: All regressions are estimated using OLS. Dependent variable: Individual test score centred with the survey year × age specic median. Standard errors appear in parathesis and are clustered by village and district-by-cohort. All estimates are adjusted for: individual and household characteristics (age, gender, birthorder, household size, mother's educational level and wealth), birth year, year, district and district-by-year fixed effects as well as birthyear district-level economic development (measured as nighttime lights). The sample excludes children born prior to 2002. Population weights applied. *** and ** denotes significance at the 1 and 5 %-level, respectively.
