# Supplementary material for: Early life malaria exposure and academic performance
Source: PLoS One. 2018 Jun 22;13(6):e0199542. doi: 10.1371/journal.pone.0199542 (PMC6014671; doi:10.1371/journal.pone.0199542)
Supplement: S5 Table — (PDF) [file pone.0199542.s013.pdf]

S5 Table Robustness: Reduced districts

	(1)	(2)	(3)	(4)	(5)	(6)
	English	English	Numeracy	Numeracy	Kiswahili	Kiswahili
Birth-year PfPR	-1.235***	-1.580***	0.526	0.561	0.293	0.353
	(0.245)	(0.288)	(0.331)	(0.351)	(0.303)	(0.324)
Observations	186,141	113,299	186,141	113,299	186,141	113,299
R-squared	0.181	0.724	0.202	0.714	0.241	0.717
Household FE	No	Yes	No	Yes	No	Yes

Notes: All regression are estimated using OLS. Dependent variable: Individual test score centered with survey year × age specic median. Standard errors appear in parathesis and are clustered by village and district-by-cohort. All estimates are adjusted for: individual and household characteristics (age, gender, birth order, household size, mother's educational level and wealth), birth year, year, district and district-by-year fixed effects as well as birthyear district-level economic development (measured as nighttime lights). The sample excludes districts where PfPR in year 2002 was greater than PfPR in 2000. Population weights applied. *** and ** denotes significance at the 1 and 5 %-level, respectively.
