# Supplementary material for: Early life malaria exposure and academic performance
Source: PLoS One. 2018 Jun 22;13(6):e0199542. doi: 10.1371/journal.pone.0199542 (PMC6014671; doi:10.1371/journal.pone.0199542)
Supplement: S6 Table — (PDF) [file pone.0199542.s014.pdf]

S6 Table Robustness: Only young and old cohorts

	(1)	(2)	(3)	(4)	(5)	(6)
	English	English	Numeracy	Numeracy	Kiswahili	Kiswahili
Birth-year PfPR	-0.929***	-0.973***	0.218	0.266	0.325	0.264
	(0.180)	(0.308)	(0.204)	(0.349)	(0.212)	(0.338)
Observations	129,056	42,821	129,056	42,821	129,056	42,821
R-squared	0.195	0.723	0.257	0.727	0.215	0.706
Household FE	No	Yes	No	Yes	No	Yes

Notes: All regressions are estimated using OLS. Dependent variable: Individual test score centered with survey year × age specic median. Standard errors appear in parathesis and are clustered by village and district-by-cohort. All estimates are adjusted for: individual and household characteristics (age, gender, birth order, household size, mother's educational level and wealth), birth year, year, district and district-by-year fixed effects as well as birth year district-level economic development (measured as nighttime lights). The sample excludes individuals born between 2002 and 2004. Population weights applied. *** and ** denotes significance at the 1 and 5 %-level, respectively.
