# Supplementary material for: Early life malaria exposure and academic performance
Source: PLoS One. 2018 Jun 22;13(6):e0199542. doi: 10.1371/journal.pone.0199542 (PMC6014671; doi:10.1371/journal.pone.0199542)
Supplement: S7 Table — (PDF) [file pone.0199542.s015.pdf]

S7 Table Robustness: Numeracy competence cutoffs

Dependent variable: Numeracy

	(1)	(2)	(3)	(4)	(5)	(6)
Panel A: Full sample						
	Numbers	Counting	Magnitudes	Addition	Subtraction	Full competancies
Birth-year PfI	0.0475*	0.0736*	0.0596	0.0660	-0.0387	-0.0862**
	(0.0244)	(0.0378)	(0.0447)	(0.0454)	(0.0402)	(0.0425)
Observations	246,325	246,325	246,325	246,325	246,325	246,325
R-squared	0.103	0.153	0.223	0.236	0.247	0.230
F-stat						
Panel B: Household fixed effects						
	Numbers	Counting	Magnitudes	Addition	Subtraction	Full competancies
Birth-year PfI	0.064	0.079	0.084	0.098*	-0.093*	-0.106*
	(0.039)	(0.056)	(0.061)	(0.058)	(0.055)	(0.059)
Observations	149,262	149,262	149,262	149,262	149,262	149,262
R-squared	0.666	0.671	0.693	0.697	0.697	0.687
F-stat						

Notes: All regressions are estimated using OLS. Dependent variable: Competencies where column (1): Dummy variable (1= Numbers or higher), column (2): Dummy variable (1=counting or higher), column (3): Dummy variable (1=Magnitudes or higher), column (4): Dummy variable (1=addition or higher), column (5): Dummy variable (1=subtraction or higher), column (6): Dummy variable (1=Full competencies). Standard errors appear in parathesis and are clustered by village and district-by-cohort. All estimates are adjusted for: individual and household characteristics (age, gender, birth order, household size, mother's educational level and wealth), birth year, year, district and district-by-year fixed effects as well as birth year district-level economic development (measured as nighttime lights). The sample excludes individuals born between 2002 and 2004. Population weights applied. *** and ** denotes significance at the 1 and 5 %-level, respectively.
