# Supplementary material for: Early life malaria exposure and academic performance
Source: PLoS One. 2018 Jun 22;13(6):e0199542. doi: 10.1371/journal.pone.0199542 (PMC6014671; doi:10.1371/journal.pone.0199542)
Supplement: S8 Table — (PDF) [file pone.0199542.s016.pdf]

S8 Table Robustness: Kiswahili competence cutoffs

Dependent variable: Kiswahili

	(1)	(2)	(3)	(4)	(5)
Panel A: Full sample					
	Letters	Words	Paragraf	Story	Full competencies
Birth-year PfPR	0.118***	0.0801*	0.00422	-0.0169	-0.0136
	(0.0394)	(0.0411)	(0.0375)	(0.0363)	(0.0371)
Observations	246,325	246,325	246,325	246,325	246,325
R-squared	0.159	0.268	0.290	0.277	0.273
F-stat					
Panel B: Household fixed effects					
	Letters	Words	Paragraf	Story	Full competencies
Birth-year PfPR	0.169***	0.117**	0.021	-0.044	-0.019
	(0.054)	(0.056)	(0.055)	(0.054)	(0.053)
Observations	149,262	149,262	149,262	149,262	149,262
R-squared	0.670	0.703	0.705	0.709	0.704
F-stat					

Notes: All regressions are estimated using OLS. Dependent variable: Competencies where column (1): Dummy variable (1= letters or higher), column (2): Dummy variable (1=words or higher), column (3): Dummy variable (1=paragraf or higher), column (4): Dummy variable (1=read story or higher), column (5): Dummy variable (1=Full competencies). Standard errors appear in parathesis and are clustered by village and district-by-cohort. All estimates are adjusted for: individual and household characteristics (age, gender, birth order, household size, mother's educational level and wealth), birth year, year, district and district-by-year fixed effects as well as birth year district-level economic development (measured as nighttime lights). The sample excludes individuals born between 2002 and 2004. Population weights applied. *** and ** denotes significance at the 1 and 5 %-level, respectively.
